# Inducing expression of ICOS-L by oncolytic adenovirus to enhance tumor-specific bi-specific antibody efficacy

**DOI:** 10.1186/s12967-024-05049-2

**Published:** 2024-03-07

**Authors:** Neshat Saffarzadeh, Emelie Foord, Eoghan O’Leary, Rand Mahmoun, Thomas Birkballe Hansen, Victor Levitsky, Thomas Poiret, Michael Uhlin

**Affiliations:** 1 Department of Clinical Science, Intervention and Technology, Karolinska Institutet, ANA Futura, Alfred Nobels Allé 8, 141 52 Huddinge, Stockholm Sweden; 2 Circio AB, Stockholm, Sweden; 3 Department of Immunology and Transfusion Medicine, Karolinska University Hospital, Stockholm, Sweden

**Keywords:** Oncolytic virus, ICOS, Bi-specific antibody, T cell, Immunotherapy

## Abstract

**Background:**

Intratumoral injection of oncolytic viruses (OVs) shows promise in immunotherapy: ONCOS-102, a genetically engineered OV that encodes Granulocyte–Macrophage Colony-Stimulating Factor (GM-CSF) demonstrated efficacy in early clinical trials, enhancing T cell infiltration in tumors. This suggests OVs may boost various forms of immunotherapy, including tumor-specific bi-specific antibodies (BsAbs).

**Methods:**

Our study investigated in vitro, how ONCOS-204, a variant of ONCOS-virus expressing the ligand of inducible T-cell co-stimulator (ICOSL), modulates the process of T cell activation induced by a BsAb. ONCOS-102 was used for comparison. Phenotypic and functional changes induced by combination of different OVs, and BsAb in T cell subsets were assessed by flow cytometry, viability, and proliferation assays.

**Results:**

Degranulation and IFNγ and TNF production of T cells, especially CD4 + T cells was the most increased upon target cell exposure to ONCOS-204. Unexpectedly, ONCOS-204 profoundly affected CD8 + T cell proliferation and function through ICOS-L/ICOS interaction. The effect solely depended on cell surface expression of ICOS-L as soluble ICOSL did not induce notable T cell activity.

**Conclusions:**

Together, our data suggests that oncolytic adenoviruses encoding ICOSL may enhance functional activity of tumor-specific BsAbs thereby opening a novel avenue for clinical development in immunotherapeutics.

**Supplementary Information:**

The online version contains supplementary material available at 10.1186/s12967-024-05049-2.

## Introduction

Tumor infiltrating lymphocytes (TILs) presence and quantity correlate with survival and decrease recurrence in cancer [1–3]. In certain cancers such as melanoma and non-small cell lung cancer, the repressed anti-tumor and proliferative functions induced by tumor cells and tumor microenvironment (TME) can be rescued by immune checkpoint blockade (ICB) [4–6]. Despite this advancement in cancer therapy many clinical trials using ICB have also highlighted their limitations as monotherapy [7, 8]. Logically, combination of multiple immunotherapies or integration of immunotherapies with other cancer treatment modalities, such as cytostatic drugs or radiation therapy have demonstrated improved outcomes compared to single-agent therapies [9]. Among these combinatory strategies, the preconditioning with oncolytic viruses (OVs) followed by bi-specific antibodies (BsAbs) has resulted in tumor regression in various tumor mouse models [10]. When replicating within tumor cells, Ovs give multiple essential advantages: (i) inducing tumor cell lysis, (ii) promoting immunogenic cell death of tumors and (iii) facilitating the spread of tumor antigen that enhance lymphocyte infiltration and provide immune cells stimulation to virus/tumor-specific epitopes/ligands. This recognition can lead to an abscopal effect and development of long-term protection through a specific antitumor memory response. Moreover, the dual-targeting capability of BsAbs allows the redirection of T cells towards tumor-specific antigens and/or overexpressed molecules. As of today, six BsAbs have received FDA approval, and many more are in clinical development [11].

In the pursuit of boosting/rescuing anti-tumor T cell responses, one approach involves targeting the inducible T-cell costimulator (ICOS), an immune checkpoint member in the CD28 superfamily, expressed by activated T cells. The engagement of ICOS with its ligand (ICOSL) leads to T cell activation and proliferation, offering the potential to augment T cell activity further. However, it's worth noting that the use of monoclonal agonists targeting ICOS, such as vopratelimab and feladilimab, have not lived up to expectations in their clinical development. This discrepancy may be partly attributed by the dual role of the ICOS ligand (ICOSL) and the ICOS costimulatory pathway in tumor immunity, where it can exhibit both anti-tumor and pro-tumor activities (especially in regulatory T cells) [12]. This duality may help explain the disappointing results and discontinuations observed in early conducted clinical trials (INDUCE-3, INDUCE-4 and ICONIC).

The OV ONCOS-102 which encodes Granulocyte–macrophage colony-stimulating factor (GM-CSF) has exhibited promising results in recent Phase I/II clinical trials for patients with advanced melanoma resistant or recurring to anti-PD-1 therapy [13]. GM-CSF enhances antitumor immunity by inducing the recruitment and maturation of different immune cells such as dendritic cells and macrophages but also T cells [14]. Yet, the precise impact of ONCOS-102 on T cell functions remains incompletely understood. Therefore, for this study, we sought to comprehensively evaluate the T cell phenotype, function, and anti-tumor activity through two distinct approaches: (i) the combination of ONCOS-102 with an EGFRxCD3 BsAb and (ii) the use of the newly engineered OV ONCOS-204, which encodes for ICOSL. These assessments were carried out in vitro, shedding light on their potential in strengthening anti-tumor T cell responses to improve efficacy in future cancer treatments.

Our study unveils a promising composition of engineered OV encoding for ICOSL and BsAb, illuminating pathways for enhanced anti-tumor T cell responses for development of a promising era in immunotherapeutics.

## Methods

### EGFR + tumor cell lines and peripheral blood mononuclear cells

EGFR + A375 malignant melanoma and A549 lung carcinoma cell lines were cultured and expanded in DMEM (Sigma-Aldrich, D6546) supplemented with 4 mM l-Glutamine (Hyclone) and 10% fetal bovine serum (FBS, Hyclone). Peripheral blood mononuclear cells (PBMCs) from buffy coat obtained from healthy donors (n = 8) were isolated using Ficoll-Hypaque density gradient (Cytiva) and cryopreserved in FBS supplemented with 10% dimethysulfoxide (Sigma-Aldrich) until use.

### Modified oncolytic adenoviruses

**Fig. 1:**
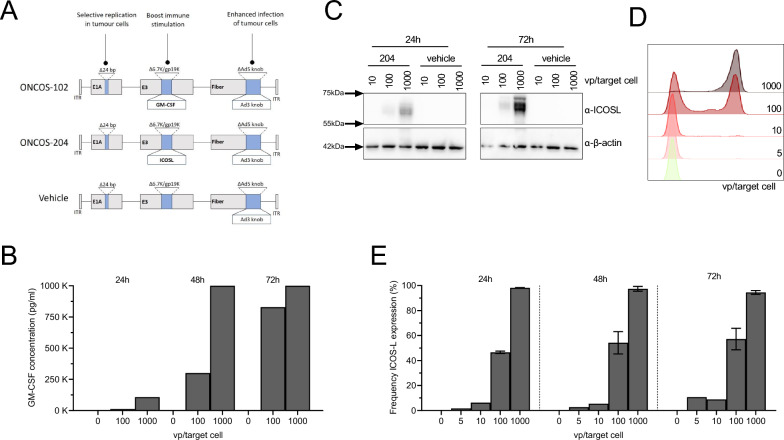
ONCOS-102 and ONCOS-204 action on tumor cells. **A** Vehicle, ONCOS-102 and ONCOS-204 plasmids design. A375 tumor cells GM-CSF production (**B**) and ICOSL production (**C**) and surface expression (**D**, **E**) 24, 48 and 72 h after infection with different concentrations of ONCOS-102 or ONCOS-204 viral particles/target cells (vp/target cells)

ONCOS-102 and ONCOS-204 are modified chimeric adenoviruses with a serotype 5 backbone, serotype 3-derived fiber knob and a 24-base pair (bp) deletion in the retinoblastoma (Rb) protein binding site of the E1A region, restricting virus replication to cancer cells. In addition, the viruses have a 965 bp deletion in the E3 region and an associated insertion of a transgene encoding either GM-CSF (ONCOS-102: Ad5/3-D24-GM-CSF, Uniprot: P04141) or ICOSL (ONCOS-204: Ad5/3-D24-ICOS-L, Uniprot: O75144), Fig. 1A. A vehicle virus (AdV5/3-D24-E3) was used as control in the current study. ONCOS-102 was produced by Biovian (Turku, Finland) and ONCOS-204 and vehicle virus were produced by OD260 Inc (ID, USA).

Details for generation of ONCOS-102 have been described previously [15] along with characteristics and use in preclinical and clinical settings [13, 16]. ONCOS-204 was constructed, amplified, and purified by OD260 Inc. using the same ONCOS-backbone but with the insertion of the 909 bp complimentary DNA (cDNA) encoding ICOSL instead of GM-CSF. The shuttle plasmid construction was done using standard cloning methods, verified by restriction analysis and sequencing, followed by cosmid construction and transfection into immortalized human embryonic kidney cells (HEK293). Virus plaques were harvested, amplified, and verified. Expression of the transgene ICOSL was confirmed by western blot (WB) before additional amplification and purification.

The generated viral stocks were aliquoted in PBS and stored at − 80 °C until use. The virus particles (vp) were used at indicated concentrations in the study.

### GM-CSF production and ICOSL expression by cancer cell lines

In a 24-well plate (AH diagnostics), human melanoma (A375) cancer cells were seeded at 50 k/ml concentration in 0,5 ml appropriate culture medium described above. Culture medium was changed 24 h later and different concentrations of OVs (vehicle, ONCOS-102 or ONCOS-204, from 0 to 1000 viral particle/target cell) were added to the culture. Supernatants and cells were collected + 24 h, + 48 h and + 72 h after the addition of different OVs. GM-CSF production was evaluated in the supernatant by ELISA following the manufacturer instructions (Mabtech AB). Cell surface ICOSL expression (A647 conjugated, clone 2D3/B7-H2, BD) was evaluated together with the surface expression of EGFR (PE conjugated, clone EMab-134, BD) after viability staining (Live/dead fixable Aqua dead cell stain, Thermo Fisher Scientific). Cells were fixed (Fixation and Permeabilization Solution, BD) before analysis by flow cytometry.

### ICOSL immunoblot

After following similar design as above, infected A375 cells were harvested in PBS and centrifuged at 1200 rpm at 4 °C for 5 min. Cell pellets were collected and resuspended in 50 μl RIPA buffer supplemented with protease inhibitors. Lysate was incubated for 20 min and then cell debris was collected by centrifugation at 12,000×*g* at 4C for 15 min. Supernatant was collected, and protein levels quantified using BCA assay (Thermo Fisher Scientific). For WB, 3ug of protein was used for analysis. Prior to WB, equal volume of 2xSDS loading buffer [125 mM Tris–HCl pH 6.8, 20% glycerol, 5% SDS, and 0.2 M DTT] was added to 3ug of protein lysate and briefly boiled at 95 °C for 5 min before loading on a 12% Tris–Glycine SDS-PAGE gel (Thermo Fisher Scientific) and run for approximately 1.5 h at 125 V. The proteins were transferred to a PVDF membrane (BioRad) by wet-blotting for 2 h at 4 °C at 30 V. Subsequently, the membrane was pre-blocked for 1 h at room temperature with 10% skim milk, followed by 1 h incubation with primary Ab (1:1,000; ab233151, Abcam) and 1 h with secondary Ab (1:10,000; ab6721, Abcam). After each Ab incubation, the membrane was rinsed 3 × 5 min in PBS + 0.05% Tween20 and 1 × 5 min wash with PBS. The protein bands were developed using SuperSignal West Femto Maximum Sensitivity Substrate kit (Thermo Fisher Scientific) and ChemiDoc XRS + (Biorad). Β-actin was used as control (primary Ab; 1:20,000; A5441, Sigma-Aldrich, and secondary Ab; 1:10,000; ab90723, Abcam).

### Titration of EGFRxCD3 bispecific antibody

A375 cancer cells were seeded in a 24-well plate, as described above to allow adherence. Twenty-four hours later, PBMCs were added at effector:target (E:T) ratio of 10:1 and different concentration of CD3xEGFR BsAb (Creative Biolab, custom-made) were added. Following 24 h co-incubation, PBMCs were collected, stained for viability and the surface markers CD3, CD4, CD8, CD25 and CD69 as listed in Additional file 1: Table S1.

### T cell phenotype and functional assay

Following the same infection design, of A375 tumor cells with the different OVs, 24 h after addition of the virus, medium was removed, and wells were washed once with PBS. PBMCs were added to each well at a 10:1 E:T ratio at 0.5 × 10^6^/ml in RPMI supplemented with 10% FBS.

For phenotype assay, EGFRxCD3 BsAb were added at suboptimal concentration of 0.02 ng/ml and co-cultured for 48 h at 37 °C. After the co-incubation, PBMCs were collected, stained for viability, and using the surface antibodies listed in Additional file 1: Table S1 and fixed (Fixation and Permeabilization Solution, BD). For each sample, no BsAb (negative) and a high concentration of BsAb (positive, 10 ng/ml) were used as controls.

For functional readout, 2 different EGFR + target cells were used: A375 and A549 tumor cell lines following the same transduction infection design and a concentration of 0.5 ng/ml of CD3xEGFR BsAb was added together with 10ug/ml Brefeldin A (Sigma-Aldrich), Golgi stop (BD Biosciences), and anti-CD107a antibody. The co-culture was kept for 6 h when PBMCs were collected, stained for viability followed by surface antibodies before fixation and permeabilization (Fixation/Permeabilization Kit from BD). PBMCs were then stained for intracellular cytokines (List of antibodies can be found in Additional file 1: Table S2). For each donor, conditions with no added BsAb, a 10 ng/ml BsAb concentration, and PMA (25 ng/ml)/ionomycin (1 μg/ml) were used as controls.

Following similar design, phenotype, and function of γδ T cells were analyzed using antibodies listed in Additional file 1: Tables S3, S4.

### Proliferation assay

Following A375 cells seeding and addition of the different OVs as described above, PBMCs were stained with cell trace violet (CTV, Thermo Fisher Scientific) following the manufacturer instructions. PBMCs were then incubated with infected A375 and 0.02 ng/ml EGFRxCD3 BsAb for 5 days. PBMCs were then collected, and the viability was assessed using the BD Horizon Fixable Viability Stain 780 (BD) before staining with CD3-PE/Cy7 (BD), CD4-FITC (BD) CD8-APC (BD) and TCRγδ-PE (Miltenyi Biotec).

### ONCOS viruses’ infectivity

A375 cells were seeded in a 24-well at 50 k/ml and infected with different OVs at 100 and 1000vp/target cells as described above. After 24 h, A375 cells were collected, pelleted, and stored in -20 °C until use. RNA extraction was conducted by adding 1 ml of TriZol (Thermo Fisher Scientific) to each sample. RNA was extracted by phenol–chloroform extraction following TriZol manufacturers guidelines (Thermo Fisher Scientific). One μg of DNase-treated total RNA was reverse transcribed using the M-MLV Reverse Transcriptase kit (Thermo Fisher Scientific) according to manufacturer’s protocol with the use of random hexamers to prime the reaction. For quantitative PCR, cDNA was mixed with Platinum SYBR Green I Master kit (Invitrogen) and ran on 7500 Fast Real-Time PCR System (Applied Biosystem). The reactions were carried out in technical triplicates. The obtained Ct values for each triplicate were transformed (2-Ct) and averaged (σ). All samples were normalized to GAPDH (i.e. E1A/GAPDH).

### Killing assay

A375 cells were seeded at 15 K cells/ml by adding 100ul per well in 96-well plate. After 24 h, different OVs were added at 100vp/target cells for 24 h before medium change and initiation of the coincubation with 15 k PBMCs (E:T ratio 10:1) and 1 ng/ml BsAb in in RPMI supplemented with 10% FBS. After 24, 48 h and 4 days con-incubation, supernatant was removed followed by a gentle wash with PBS before addition of DMEM (Sigma-Aldrich, D6546) supplemented with 4 mM L-Glutamine (Hyclone) and 10% fetal bovine serum (FBS, Hyclone). and CellTiter 96® AQueous One Solution Cell Proliferation Assay (Promega) following manufacturer’s instructions. Controls were medium only, virus infection only and no BsAb addition. Absorbance at 490 nm was recorded after 2 h incubation using CLARIOstar multireader (BMG Labtech). PBMCs + BsAb specific killing was calculated using the absorbance (Abs) as follows:

$${\text{{\%}\,specific \, killing}}=100\times \left(1-\frac{Abs\, of\, PBMCs+BsAb+OV-Abs\, of\, OV \,only}{Abs\, of\, PBMCs+BsAb}\right)$$

### Intracellular expression of ICOS

Intracellular ICOS was evaluated after the co-incubation at 10:1 E:T ratio with A375 cancer cells infected with ONCOS-204 at 100 vp/target cells. Briefly, PBMCs were collected after 24 h co-incubation with target cells and 0.05 ng/ml EGFRxCD3 BsAb, washed with PBS and stained for viability followed by surface antibodies CD3-PE/Cy7 (Biolegend), CD4-FITC (BD), CD8-APC/Cy7 (BD) and CD69-APC (Biolegend). Intracellular staining with ICOS-BV785 (Biolegend) was performed for 30 min after using Fixation/Permeabilization Kit (BD).

### ICOSL expression on effector T cells

PBMCs were harvested after 24 h co-incubation with ONCOS-204 infected A375 cancer cells and 0.05 ng/ml EGFRxCD3 BsAb. After washing, and staining with viability dye, PBMCs were stained with EGFR-PE (BD), CD3-PerCP (Biolegend), ICOSL-A647 (BD), CD4-A700 (BD), CD8-APC/Cy7 (BD), ICOS-BV421 (BD) and CD69-BC785 (Biolegend).

### ICOSL plasmid transfection

Transfection of 25 k A375 tumor cells with ICOSL plasmid (2ug DNA) was performed using the Lipofectamine™ 3000 Transfection Reagent kit (ThermoFisher Scientific) following the manufacturer’s instructions.

### Soluble ICOSL production and quantification

Different concentrations of ONCOS-204 were added to A375 tumor cells. Supernatants were collected after 48 h and stored at − 20 °C until use. Soluble ICOSL was quantified using the Human B7-H2/ICOS Ligand ELISA Kit (Invitrogen) following the manufacturer’s instructions.

For western blotting, supernatant was collected and centrifuged at 1200 rpm at 4 °C for 10 min to remove cell debris. Following centrifugation, 20 µl of supernatant was mixed with equal volume of 2xSDS loading buffer and briefly boiled at 95 °C. For western analysis, 35 µl of each sample was loaded and western blotting was conducted as described above.

### Data acquisition and statistics

All cell acquisition was performed by CytoFlex flow cytometer (Beckman Coulter). Flow cytometry data were data analyzed using FlowJo V.10.8 Software (BD Life Sciences). Graphical representation and statistical analysis were performed using Prism 9 (GraphPad Software). For analysis, percentage change was calculated as follows:

$$\%change=\frac{value\, of\, interest}{value\, with\, BsAb, no\, Adv}\times 100-100$$

Comparisons of paired samples exposed to different conditions (OVs or presence of BsAb) were performed using Student t test or Friedman test with Dunn’s correction. Comparisons of paired samples of two groups were performed using Wilcoxon matched- pairs signed rank test. The p-value was set at p < 0.05.

### Data availability

All data supporting the current study are presented within the article. Data related to this study may be requested from the authors.

## Results

### Dose- and time-dependent activity of ONCOS-102 and ONCOS-204 on cancer cell lines

ONCOS-102 and ONCOS-204 share a similar design (Fig. 1A). ONCOS-102 induced high GM-CSF production by the human melanoma A375 cancer cells, evident within 24 h post-infection at 100 virus particles/target cell (vp/target cell, Fig. 1B). The newly generated OV ONCOS-204 induced protein ICOSL production (Fig. 1C), with expression levels evaluated across various ONCOS-204 concentrations. Notably, ICOSL was rapidly and consistently expressed on the surface of A375 tumor cells for at least 72 h post-infection (Fig. 1D, E).

### Titration of EGFR-CD3 bi-specific antibody to achieve sub-optimal activation

**Fig. 2:**
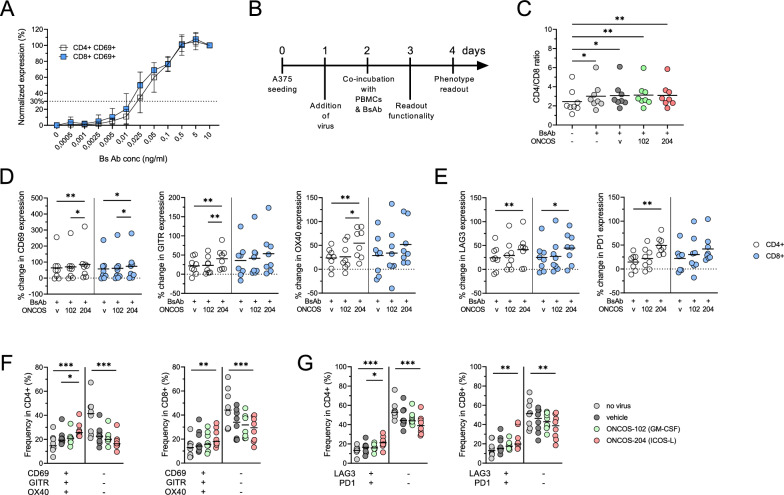
Phenotype changes induced by EGFRxCD3 BsAb and OVs. **A** Titration of EGFRxCD3 BsAb by the activation (CD69 expression) of CD4 + and CD8 + T cells (n = 3). **B** Experimental design. **C** CD4/CD8 ratio of T cells after 48 h incubation with target cells in different conditions: presence or absence of BsAb and different A375 tumor cell infections (v: vehicle, ONCOS-102 or ONCOS-204). **D** Percentage change of expression of CD69, GITR and OX40 in CD4 + (white) and CD8 + (blue) T cells upon exposure to infected A375 tumor cells. **E** Percentage change of expression of LAG-3 and PD-1 in CD4 + (white) and CD8 + (blue) upon exposure to infected A375 tumor cells. **F** CD69, GITR and OX40 co-expression analysis in CD4 + and CD8 + T cells in different conditions: non-infected tumor cells (no virus) vs. tumor cells infected with vehicle or ONCOS-102 or ONCOS-204. **G** LAG-3 and PD-1 co-expression analysis in CD4 + and CD8 + T in different conditions: non-infected tumor cells (no virus) vs. tumor cells infected with vehicle or ONCOS-102 or ONCOS-204. Friedman test with Dunn’s correction was used (**C**–**G**) to compare groups of paired samples exposed to different conditions (n = 8). *p < 0.05, **p < 0.01, ***p < 0.001. Medians are represented

To evaluate the specific impact of virus-induced payloads produced by the tumor cells on T cells, we titrated the concentration of EGFRxCD3 BsAb. We aimed for a suboptimal activation level (i.e. 30% of maximum stimulation) by testing different concentrations. After 48 h, we found that 0.02 ng/ml was suitable, as indicated by CD69 expression on the T cell surface (Fig. 2A). This suboptimal activation was chosen to ensure enough T cell activation by BsAb while allowing for additional co-stimulation from virus-expressed substances in subsequent experiments. Throughout the paper, we used this dose unless specified otherwise.

### Phenotypical changes induced by EGFRxCD3 bi-specific antibody are further amplified by ONCOS-102 and ONCOS-204

PBMCs supplemented with BsAb were co-incubated with A375 tumor cells infected with different concentrations of vehicle, ONCOS-102 or ONCOS-204 viruses as described (Fig. 2B). After 48 h of co-incubation, T cell phenotype was evaluated. The addition of suboptimal concentration of EGFRxCD3 BsAb increased the CD4/CD8 ratio and expression of activation markers (4-1BB, CD25, CD69, GITR and OX40) regardless of the infection status of the target cells (p < 0.05, compared to control condition with no BsAb, no OVs, Fig. 2C and Additional file 2: Fig. S1A, B).

Comparing the impact of the different OVs on T cells revealed that ONCOS-204-infected tumor cells, expressing ICOSL, induced the highest proportion of CD4 + T cells expressing activation markers CD69, GITR and OX40 (p < 0.01, Fig. 2D and Additional file 2: Fig. S1C). For CD8 + T cells, this increase was significant only for CD69 (p < 0.05, Fig. 2D and Additional file 2: Fig. S1C). Additionally, ONCOS-204 increased the proportion of T cells expressing LAG-3 and PD-1 compared to the vehicle virus and ONCOS-102 (p < 0.05, Fig. 2E and Additional file 2: Fig. S1D). Consequently, co-expression of activation markers CD69, GITR and OX40 were found at significantly higher proportions among CD4 + and CD8 + T cells exposed to ONCOS-204-infected tumor cells (p < 0.05, Fig. 2F). Similarly, the proportion of cells co-expressing LAG-3 and PD-1 were higher among CD4 + and CD8 + T cells exposed to ONCOS-204 infected target cells compared to the other conditions (p < 0.05, Fig. 2G).

Overall, the infection of target tumor cells with OVs leading to the expression of ICOSL, activates CD4 + and CD8 + T cells as indicated by the increased expression of CD69, GITR, OX40 as well as the immune checkpoint markers LAG-3 and PD-1.

### Function and proliferation of BsAb activated T cells are further enhanced by ONCOS-102 and ONCOS-204

To examine the functional effects induced by ONCOS-102 and ONCOS-204, we initially investigated the response solely induced by the EGFRxCD3 BsAb used at a high concentration of 10 ng/ml (Additional file 2: Fig. S2A). Results indicated that the BsAb primarily increased the frequency of degranulating (CD107a +) and TNF-producing T cells (median up to 30%) while modest increases were observed in IFNγ-producing T cells (median up to 15% of CD8 + and 3% of CD4 + T cells, respectively). For the 6 h functional assay, a BsAb concentration of 0.5 ng/ml was used to better observe changes induced by ONCOS-102 and ONCOS-204 infection.

**Fig. 3:**
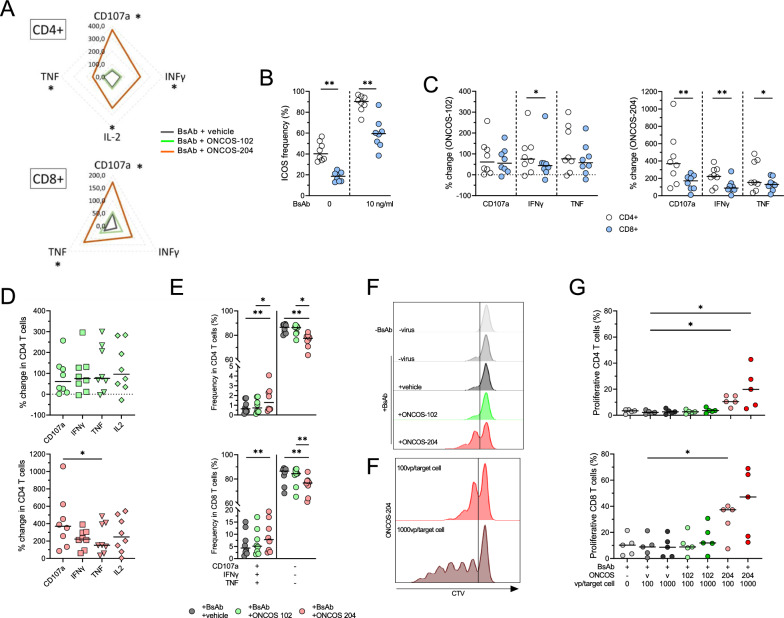
Functional changes induced by EGFRxCD3 BsAb and OVs. **A** Radar plot of the frequency of CD107a + , IFNγ, TNF + and IL-2 + CD4 + and CD8 + T cells after co-culture with A375 tumor cells infected with different virus. **B** Frequency of ICOS expression on CD4 + and CD8 + T cells induced by incubation of no or 10 ng/ml EGFRxCD3 bsAb and A375 target cells. **C** Percentage change of cytokines producing (TNF and IFNγ) and CD107a expressing T cells between CD4 + and CD8 + after co-incubation with tumor cells infected with ONCOS-102 (left) or ONCOS-204 (right). **D** Differences between change of CD107a expressing and cytokines producing (IL-2, TNF and IFNγ) CD4 + T cells induced by ONCOS-102(top, green) or ONCOS-204 (bottom, red) infected tumor cells. **E** CD107a, IFNγ and TNF co-expression analysis in CD4 + (top) and CD8 + (bottom) T cells in different conditions: non-infected tumor cells (no virus) vs. tumor cells infected with vehicle or ONCOS-102 or ONCOS-204. Representative histogram of T cells proliferation (**F**) and proportion of proliferative CD4 + (top) and CD8 + (bottom) T cells (**G**) after 10 days of incubation with target cells in different conditions: presence BsAb and the different A375 tumor cell infections (v: vehicle, ONCOS-102 or ONCOS-204) and viral concentration (100 vs. 1000 vp/target cells), n = 5. **A**–**E**, n = 8. Friedman test with Dunn’s correction was used to compare the groups of paired samples exposed to different conditions. *p < 0.05, **p < 0.01, ***p < 0.001. Medians are represented

Overall, the addition of OVs increased the frequency of degranulating and cytokine-producing CD4 + and CD8 + T cells (Additional file 2: Fig. S2B, C). Normalizing the data using the condition without virus (only BsAb, no OV) revealed that ONCOS-204-infected target cells augmented the proportion of T cells expressing CD107a, TNF, IFNγ (and IL-2 for CD4 + T cells) compared to virus vehicle and ONCOS-102-infected target cells (p < 0.05, Fig. 3A). Observing the basal ICOS expression and expression after BsAb stimulation were around 20% higher among CD4 + compared to CD8 + T cells (p < 0.01, Fig. 3B), we investigated how this translated to functionality. Consistent with earlier findings, ONCOS-204 infection of target cells more significantly enhanced the functionality of CD4 + T cells compared to CD8 + T cells (p < 0.05, Fig. 3C). The proportion of IFNγ-producing CD4 + T cells was also more prominently increased by the exposure to ONCOS-102-infected target cells compared to CD8 + T cells (p < 0.05).

The function of CD4 + or CD8 + T cells remained largely unaffected by exposure to the vehicle virus or ONCOS-102 (Fig. 3D and Additional file 2: Fig. S2D, E). In contrast, degranulation (CD107a +) of CD4 + T cells was significantly increased when exposed to ONCOS-204-infected target cells (median 370% increase, p < 0.05, Fig. 3D). In line with the observed phenotypical changes, T cells (CD4 + and CD8 +) exposed to ONCOS-204-infected target cells exhibited the highest multifunctional proportion among the conditions (CD107a + IFNγ + TNF + , p < 0.05, Fig. 3E) while T cells exposed to vehicle virus-infected target cells showed the highest inactive proportion (p < 0.01).

In addition, we investigated the T cell functional effects induced by infection of A549 lung cancer cells line by ONCOS-102 and ONCOS-204 (Additional file 2: Fig. S3A–D). A549 exhibited a higher level of EGFR expression than A375 cells line (Additional file 2: Fig. S3A). At 100vp/target cell, ONCOS-204 induced ICOS-L surface expression comparable to A375 (Additional file 2: Fig. S3B). Similar to the observation made using A375 cells, CD4 + and CD8 + T cell functions were increased by exposure to the ONCOS-204-infected A549 tumor cells as compared to vehicle virus or ONCOS-102 (Additional file 2: Fig S3C, D).

Furthermore, T cell proliferation mirrored the functional changes, depending on the stimuli induced by the different infection of target cells (Fig. 3F). CD8 + T cells demonstrated a higher proliferative fraction (median 47%) than CD4 + T cells (median 20%). However, both CD4 + and CD8 + T cells exposed to ONCOS-204-infected target cells displayed a higher proliferative fraction in a virus dose-dependent manner (p < 0.05, Fig. 3G).

To summarize, the exposure to ONCOS-204-infected target tumors expressing ICOSL significantly increased the functional activity and proliferation of T cells. These effects were most pronounced in the CD4 + T cell subset.

### Modified OVs do not impact γδ T cells phenotype to the same extent as conventional αβ T cells.

In addition to conventional αβ T cells, we explored the impact of combining EGFRxCD3 BsAb with different OVs on γδ T cells, constituting approximately 1–10% of peripheral blood T cells. Similar to conventional T cells, the BsAb effectively stimulates γδ T cells proportionally to the concentration used (0.5 vs 10 ng/ml, Additional file 2: Fig. S4A). Overall, the infection of target cells infected with various OVs, with or without addition of BsAb, did not affect the frequency of γδ T cells or the proportion of Vδ1 or Vδ2 subsets (Additional file 2: Fig. S4B, C). No change in the expression of CD69 was observed under any conditions, and only the addition of BsAb increased the frequency of TNF-, IFNγ- and MIP-1β-producing γδ T cells with no additional enhancement attributed to specific viral-derived stimuli (Additional file 2: Fig. S4D, E). There was a trend for increased proliferation of γδ T cells when co-incubated with ONCOS-204-infected target cells (p = 0.054, Additional file 2: Fig. S4F).

### Killing potency of T cells is enhanced by ONCOS-102 and ONCOS-204

**Fig. 4:**
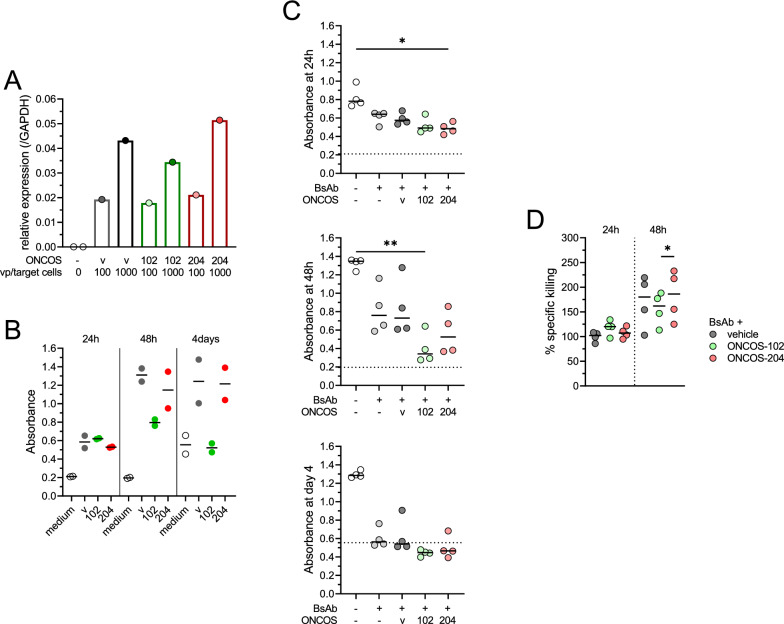
Cytotoxic activity of T cells in combination with EGFRxCD3 BsAb and oncolytic viruses. **A** Viruses infectivity quantification of A375 tumor cells by RT-qPCR. **B** Overtime (24 h, 48 h, 4 days) A375 tumor cells viability exposed to 100vp/target cells of vehicle (v), ONCOS-102 or ONCOS-204 virus, assessed by absorbance (490 nm). n = 2 repeats. **C** A375 tumor cells viability exposed to 100vp/target cells of vehicle, ONCOS-102 or ONCOS-204 virus. Viability assessed 24 h, 48 h and 4 days after PBMCs and BsAb addition. Dot horizontal line corresponds to the medium background absorbance. **D** specific PBMCs cytotoxic capacity induced by BsAb and vehicle, ONCOS-102 or ONCOS-204 virus. **C**, **D**, n = 4. Friedman test with Dunn’s correction was used to compare the groups of paired samples exposed to different conditions. *p < 0.05, **p < 0.01. Medians are represented

To assess the cytotoxic capacity of T cells, considering the increased function and proliferation observed in the ONCOS-204 condition, we conducted experiments with a similar design. We ensured that the different OVs had similar infectivity (Fig. 4A) and evaluated their sole tumor oncolytic ability by monitoring A375 cells viability overtime after OVs infection. While no significant reduction in viability observed after 24 h, ONCOS-102 exhibited stronger oncolytic capacity compared to the vehicle virus and ONCOS-204 at a similar vp/target cell concentration at 48 h and 4 days after infection (Fig. 4B). After 24 h co-incubation of infected A375 cells with PBMCs and EGFRxCD3 BsAb, the viability of tumor cells was most decreased in the ONCOS-204 condition compared to the control condition (no BsAb, no virus, p < 0.05, Fig. 4C). After 48 h, A375 cells in the ONCOS-102 condition appeared to have most reduced viability as compared to the control condition (p < 0.01). By day 4, the median viability of conditions with BsAb, regardless of OV infections reached background levels, suggesting the absence of viable tumor cells (Fig. 4C).

No difference in specific killing attributed to T cells and BsAb were observed between the different OVs condition after 24 h co-incubation. However, after 48 h, T cells in combination with BsAb incubated with ONCOS-204-infected tumor cells showed higher specific killing compared to when incubated with ONCOS-102-infected tumor cells (p < 0.05, Fig. 4D).

In summary, while ONCOS-102 demonstrated superior oncolytic activity compared to the vehicle OV and ONCOS-204, ONCOS-204-infected tumor cells enhanced the killing capacity of T cells via the EGFRxCD3 BsAb interaction more than ONCOS-102-infected tumor cells.

### ONCOS-204 induced soluble production of ICOSL

**Fig. 5:**
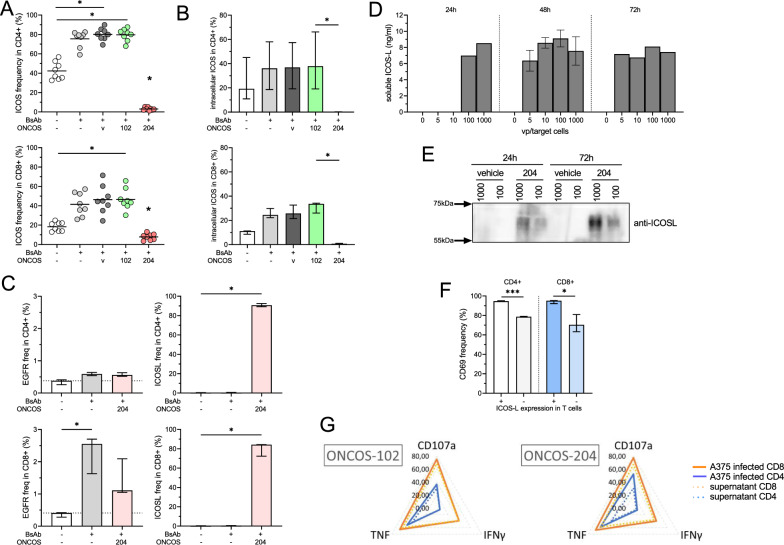
ONCOS-204 induced broad co-stimulation by the soluble production of ICOSL. **A** Surface ICOS expression on CD4 + (top) and CD8 + (bottom) T cells after 48 h incubation with target cells in different conditions: presence or absence of BsAb and the different A375 tumor cell infections (v: vehicle, ONCOS-102 or ONCOS-204), n = 8. **B** intracellular ICOS expression on CD4 + (top) and CD8 (bottom) T cells after 48 h incubation with target cells in different conditions. **C** Surface expression of EGFR (left) and ICOSL (right) on CD4 + (top) and CD8 (bottom) T cells after 48 h incubation with target cells in different conditions (presence of BsAb and ONCOS-204). Quantification of soluble ICOSL concentration in supernatant (in ng/ml, **D**) and WB of sICOL presence (**E**) in supernatants 24, 48 and 72 h after infection of A375 with different concentrations of ONCOS-204 (0, 5, 10, 100 and 1000 vp/target cells). **F** Surface expression of CD69 on ICOSL + and ICOSL- population of CD4 + and CD8 + T cells. **G** Radar plot of the frequency of CD107a + , IFNγ and TNF + CD4 + and CD8 + T cells after co-culture with A375 tumor cells infected with virus or A375 tumor cells and supernatant issue from virus-infected A375 cells. Student t test or Friedman test with Dunn’s correction was used to compare the two conditions or groups of paired samples exposed to different conditions, **B**, **C**, **F**, **G**; n = 3. *p < 0.05

We previously demonstrated that ONCOS-204 induces ICOSL expression at the surface of the A375 tumor cells in a time- and concentration-dependent manner (Fig. 1D). Consistent with the earlier phenotype changes (Additional file 2: Fig. S1A), the addition of the EGFRxCD3 significantly increased BsAb surface ICOS expression (Figs. 3C and 5A). Interestingly, minimal expression of ICOS was observed when CD4 + and CD8 + T cells were co-cultured with target cells infected with ONCOS-204 (p < 0.05, Fig. 5A). A similar observation was made when target cells were transfected with an ICOSL plasmid before addition of T cells and BsAb co-culture; a decrease of ICOS surface expression was noted among CD4 + and CD8 + T cells exposed to ICOSL plasmid-transfected tumor cells, and although T cell cytotoxic function was improved, it was not statistically significant (Additional file 2: Fig. S5A, B).

To validate ICOS/ICOSL-dependent activation, target cells were infected with various concentrations of ONCOS-204 before co-incubation with T cells and BsAb. As anticipated, the increase in the proportion of T cells expressing CD69 was directly proportional to viral particle concentration (Additional file 2: Fig. S5C). Conversely, the surface ICOS expression, assessed with two different anti-ICOS antibodies (clones C398.4A and Dx29), decreased with the concentration of ONCOS-204 (p < 0.05, Additional file 2: Fig. S5C).

We hypothesized that ICOSL/ICOS interaction might induce the internalization of ICOS molecules from the cell surface. To investigate this, we assessed the presence of intracellular ICOS after co-culture of T cells in different conditions. Although evident activation induced by BsAb and different co-stimuli, no intracellular ICOS molecules were observed among CD4 + or CD8 + T cells when co-incubated with ONCOS-204-infected target cells (Fig. 5B, Additional file 2: Fig. S5D). Consequently, internalization of ICOS upon engagement was ruled out as an explanation for the absence of ICOS at the cell surface upon ONCOS-204 exposure.

To investigate the possibility for the process of trogocytosis—a phenomenon where molecules from target cells are harvested and then expressed by the effector cells—we assessed the expression of EGFR and ICOSL at the T cell surface (Fig. 5C). We observed the presence of EGFR molecules on the surface of a proportion of CD8 + T cells after co-incubation with target cells and EGFRxCD3 BsAb (p < 0.05) suggesting that some degree of trogocytosis did occur. Interestingly, no significant EGFR expression was observed among CD4 + T cells nor in the ONCOS-204 condition. Yet, exclusive, and high expression of ICOSL (median > 80%, p < 0.05) was observed at the surface of both CD4 + and CD8 + T cells when co-incubated with target cells infected with ONCOS-204 suggesting that ICOSL presence on the surface of T cells was not caused by trogocytosis.

To explore further, the production of soluble ICOSL (sICOSL) by target cells infected with ONCOS-204 was investigated. Interestingly, high levels of sICOSL were observed for at least 72 h at different viral particle concentrations (Fig. 5D, E). Since higher proportions of CD69 + CD4 + and CD8 + T cells were found in the T cell fraction that expressed ICOSL at the surface (Fig. 5F), we questioned the single impact of soluble ICOSL on T cells activity and function. For this purpose, we transferred supernatant from virus-infected A375 cells (vehicle, ONCOS-102 and ONCOS-204) to a co-culture of T cells and target cells that were not infected by OVs before assessing the proportion of T cells expressing CD107a, IFNγ and TNF. As expected, no difference in T cell function was observed between the two conditions involving vehicle virus and ONCOS-102 (i.e. co-cultured with A375 cells infected with virus versus supernatant from virus-infected A375 cells, Fig. 5G and Additional file 2: Fig. S5E). On the other hand, the transfer of supernatant containing sICOSL from ONCOS-204-infected A375 cells did not show a T cell functionality equivalent to T cells that were co-cultured with A375 with surface ICOSL surface expression (Fig. 5G).

Altogether, these data identified a high secretion of sICOSL induced by ONCOS-204 infection of the tumor cells, but sICOSL did not impact T cell function in our setting, suggesting that ONCOS-204 would have a local and intratumoral effect on T cell function.

## Discussion

Different strategies of immunotherapy have revolutionized cancer treatment, yet their effectiveness often faces limitations in ensuring long-term patient survival when used alone. This study delved into the potential of combining different OVs with EGFRxCD3 BsAb in vitro, focusing on patients with EGFR + tumor cells like melanoma, brain cancer or NSCLC. Our findings demonstrate a significant enhancement in T cell function and cytotoxicity when exposed to ONCOS-204-infected tumor cells combined with BsAb activation.

While Talimogene laherparepvec (T-VEC), the sole FDA-approved oncolytic therapy encoding GM-CSF, has proven effective for advanced melanoma [17], challenges such as TME, delivery strategies, and host-developed neutralizing antibodies impede the broader clinical use of T-VEC and other OVs in development. Treatment strategies exploring OVs either as neoadjuvant [18] and/or in combination with other therapies such as checkpoint inhibitors are currently being expanded [13, 19]. In this study, we highlight how pre-conditioning tumor cells with various OVs (AdV5/3-D24-E3-based) enhanced T cell activation and function when combined with BsAb. Combining OVs with BsAbs has proven effective, either by preconditioning the TME with the virus followed by BsAb treatment [10] or using OVs encoding T cell engagers or BsAbs, resulting in increased T cell functionality and stronger anti-tumor activity [20, 21]. Preconditioning with ONCOS-102 before EGFRxCD3 BsAb and T cell co-culture demonstrated improved T cell activation and functionality. The transfer of ONCOS-102-infected tumor medium to the functional assay between uninfected tumor cells with BsAb and T cells demonstrated the potential systemic effects of GM-CSF production by ONCOS-102-infected tumor cells, as observed in a humanized NOG mouse model [22].

Furthermore, our study reveals that preconditioning with ONCOS-204 outperformed ONCOS-102 in terms of T cell activation, functionality, proliferation, and T cell cytotoxicity. This distinction in T ell engagement is likely attributed to the different mechanisms of action induced by the expressed payloads and selection of readouts and time points. The ICOSL/ICOS immune complex, known for its multifaceted functions, activates CD8 and CD4 T cells with anti-tumor properties but also controls regulatory T cells (Treg) homeostasis [23]. Our study found that ONCOS-204-induced ICOSL/ICOS complex significantly improved T cell proliferation and cytotoxic activity, suggesting that the impact of ICOS + Treg cells, if present, was minimal. Nevertheless, further investigation into the ONCOS-204 effect on ICOS + Treg cells is warranted, as higher proportions of Tregs and ICOS + Tregs have been observed in the TME [24].

The impact of ONCOS-204-infected tumor cells on CD4 T cells appeared more pronounced than on CD8 T cells. Highlighting the higher inherent ICOS-L surface expression on CD4 T cells. Unlike CD8 T cell co-stimulation that is primarily driven by the engagement of CD28 and 4-1BB molecules, CD4 T cell co-stimulation is also largely dependent on the ICOS-L/ICOS pathway [25, 26]. Even while the role of ICOS pathway is less well described in CD8 T cells, there are data elucidating that its overexpression elevates CD8 T cell expansion and function during *Listeria* infection [27]. In concordance with this, we also showed that the ICOS-L/ICOS interaction induced by ONCOS-204 enhanced the cytotoxic profile and proliferation of CD8 T cells. The ICOSL/ICOS interaction is also known to be critical to the development of the Th2 [28], and Th17 [29] immunity, as well as different memory subsets [23]. Notably, our study unveils that CD4 T cells influenced by ONCOS-204 exhibited a higher cytotoxic capacity with increased degranulation and the production of Th1 cytokines like TNF and IFNγ. These effects present multiple anti-tumor effects by inhibiting/killing tumor growth and modulating the immune response to enhance anti-tumor activity [1, 30]. Further characterization of the impact of ONCOS-204 on T cell memory subsets, and specific CD4 T cell subsets such as Th1, Th2, and Th17 would be valuable and investigating the implications in a tumor model context.

The presence of soluble ICOSL produced by ONCOS-204 infected tumor cells also requires further investigation. The question whether sICOSL or membrane-bound ICOSL is responsible for the loss of surface and intracellular ICOS on T cells is still not fully resolved by our data. In our setting, T cells exposed to ONCOS-204-infected tumor cells displayed a highly activated phenotype and increased function. These changes were not induced by the transfer of sICOSL loaded supernatant. A possible conclusion would be that the soluble form may lack immunostimulatory function, suggesting that the absence of T cells activation may reduce the risk of unwanted “off-site” ICOSL/ICOS-dependent T cell activation generated by ONCOS-204 therapy. Increased plasma sICOSL has been observed during active lupus, compared to inactive one suggesting an immunopathological role of sICOSL [31]. Therefore, assessing the impact of sICOSL on activated T cells, known to increase their ICOS surface expression would be interesting. Furthermore, the impact of ONCOS-204-infected cells causing the expression of ICOSL and production of sICOSL in the tumor microenvironment should be extended beyond T cells to encompass a broader spectrum of immune cells such as NK cells with a focus on understanding their role in generating antibody-dependent cellular cytotoxicity and NK-specific cytotoxicity functions remains to be investigated.

Surprisingly, aside from trending increase in proliferation, there were no apparent phenotypical or functional changes observed among γδ T cells when triggered by OVs. In our study, using suboptimal concentrations of BsAb, the proportion of γδ T cells was approximately 5%, it is therefore possible that the dominant presence of conventional T cells may have mobilized majority of available BsAb, leading to an underestimation of the effect of OVs on the γδ T cells. Notably, ICOSL/ICOS interaction among γδ T cells has been implicated in the development of IL-17A-producing γδ T cells [32]. Therefore, a more in-depth investigation into the impact of ONCOS-204 on intratumoral γδ T cells and their various subsets in cancer patients is needed.

## Conclusion

Our study highlights an encouraging potential of combining OVs, specifically ONCOS-204, with EGFRxCD3 BsAbs to enhance T cell activation and cytotoxicity in the context of EGFR + tumor cells, such as melanoma. The superior performance of ONCOS-204 over ONCOS-102 in promoting T cell function by modulating the response through the ICOSL/ICOS pathway underscores the complexity and significance of leveraging OVs in combination therapies. Further investigations into the role of ICOS + Treg cells, the impact of sICOSL, and the response of γδ T cells will contribute to refining the potential therapeutic strategies.

### Supplementary Information

**Additional file 1.** Supplementary Tables S1–S4 and Legends for Supplementary Figures S1–S5

**Additional file 2.** Supplementary Figures S1–S5

## Data Availability

Data generated and used and/or analyzed during are available from the corresponding author on reasonable request.

## Abbreviations

TILs: Tumor infiltrating lymphocytes
TME: Tumor microenvironment
ICB: Immune checkpoint blockade
OVs: Oncolytic viruses
BsAbs: Bi-specific antibodies
ICOS(L): Inducible T-cell costimulatory (ligand)
GM-CSF: Granulocyte–macrophage colony-stimulating factor
EGFR: Epithelial growth factor receptor
FBS: Fetal bovine serum
Bp: Base pair
Rb: Retinoblastoma
cDNA: Complimentary DNA
WB: Western blot
Treg: Regulatory T cells
sICOSL: Soluble ICOSL
