# Supplementary material for: Inducing expression of ICOS-L by oncolytic adenovirus to enhance tumor-specific bi-specific antibody efficacy
Source: J Transl Med. 2024 Mar 7;22:250. doi: 10.1186/s12967-024-05049-2 (PMC10921603; doi:10.1186/s12967-024-05049-2)
Supplement: Supplementary file 1 — Additional file 1. Supplementary Tables S1–S4 and Legends for Supplementary Figures S1–S5 [file 12967_2024_5049_MOESM1_ESM.pdf]

Additional file

Inducing expression of ICOS-L by oncolytic adenovirus to enhance tumor-specific bi-specific antibody efficacy

Neshat Saffarzadeh[1], Emelie Foord[2], Eoghan O'Leary[1, 2], Rand Mahmoun[1], Thomas Birkballe Hansen[2], Victor Levitsky[2], Thomas Poiret[1] *, Michael Uhlin[1,3] *

[1]Department of Clinical Science, Intervention and Technology, Karolinska Institutet, Stockholm, Sweden. [2]Circio AB, Stockholm, Sweden [3]Department of Immunology and Transfusion Medicine, Karolinska University Hospital, Stockholm, Sweden

*Contributed equally

Corresponding author: Thomas Poiret, Karolinska Institutet, ANA Futura, Alfred Nobels Allé 8, 141 52 Huddinge. Email: thomas.poiret@ki.se

Additional file Tables

Color	Marker	Company	Clone
FITC	PD-1	BD Biosciences	MIH4
PE	41BB	BD Biosciences	4B4-1
PE/Dazzle 594	OX40	Biolegend	Ber-ACT35 (ACT35)
PerCP	CD3	Biolegend	UCHT1
PE Cy7	CD25	BD Biosciences	2A3
APC	CD69	Biolegend	FN50
A700	CD4	BD Biosciences	RPA-T4
APC Cy7	CD8	BD Biosciences	SK1
Bv421	GITR	BD Biosciences	V27-580
	DCM Aqua	Thermofisher	N.A.
Bv650	LAG-3	Biolegend	11C3C65
Bv785	ICOS	Biolegend	C398.4A

Table S1. T cell phenotype panel

Color	Marker	Company	Clone
FITC	CD4	BD Biosciences	RPA-T4
PE	CD107a	BD Biosciences	H4A3
PE/eFluor610	TNF	Thermofisher	MAb11
PE Cy7	CD3	Biolegend	HIT3a
APC	INFγ	BD Biosciences	4S.B3
A700	IL-2	Biolegend	MQ1-17H12
APC Cy7	CD8	BD Biosciences	SK1
	DCM Aqua	Thermofisher	N.A.

Table S2. T cell functional panel (intracellular cytokine staining)

Color	Marker	Company	Clone
FITC	Vd1	Thermofisher	TS8.2
PE	TCR gd	Miltenyi Biotec	REA591
PE-CF594	CCR7	BD Biosciences	150503
PerCP	CD3	Biolegend	UCHT1
PE Vio770	CD27	Miltenyi Biotec	M-T271
APC	CD45RO	BD Biosciences	UCHL1
A700	CD4	BD Biosciences	RPA-T4
APC Cy7	CD8	BD Biosciences	SK1
Vioblue	Vd2	Miltenyi Biotec	123R3
	DCM aqua	Thermofisher	N.A.
Bv650	NKG2D	BD Biosciences	1D11
Bv785	DNAM-1	Biolegend	11A8

Table S3 γδ T cell phenotype panel

Color	Marker	Company	Clone
FITC	Vd1	Thermofisher	TS8.2
PE	TCR gd	Miltenyi Biotec	REA591
PerCP-Cy5.5	IL-17a	BD Biosciences	N49-653
PE Cy7	IFNg	BD Biosciences	B27
APC	TNF	BD Biosciences	MAb11
ALEXA700	MIP-1b	BD Biosciences	D21-1351
APC Cy7	Viability	BD Biosciences	N.A.
VioBlue	Vd2	Miltenyi Biotec	123R3
Bv510	CD3	BD Biosciences	UCHT1
Bv650	NKG2D	BD Biosciences	1D11
Bv785	CD69	BD Biosciences	FN50

Table S4. γδ T cell functional panel (intracellular cytokine staining)

Additional file Figures

Figure S1

Surface expressions of 4-1BB, CD25, CD69, GITR and OX40 in CD4+ (**A**) and CD8+ (**B**) exposed to different conditions: presence or absence of BsAb and the different A375 tumor cell infections (v: vehicle, ONCOS-102 or ONCOS-204). **C.** Percentage change of expression of 4-1BB and CD25 in CD4+ (white) and CD8+ (blue) T cells upon exposure to infected A375 tumor cells. **D.** Surface expression of LAG-3 and PD-1 in CD4+ and CD8+ T cells exposed to different conditions: presence or absence of BsAb and the different A375 tumor cell infections (v: vehicle, ONCOS-102 or ONCOS-204). Friedman test with Dunn's correction was used to compare the groups of paired samples exposed to different conditions (n=8). *p<0.05, **p<0.01, ***p<0.001. Medians are represented.

Figure S2

A. Frequency of CD107a, IFNγ, TNF and IL-2 positive CD4+ (top) and CD8+ (bottom) T cells induced by 6h incubation of 10ng/ml EGFRxCD3 BsAb and A375 target cells. Frequency of CD107a, IFNγ, TNF and IL-2 positive CD4+ (**B**) and CD8+ (**C**) T cells exposed to different conditions: presence or absence of BsAb and the different A375 tumor cell infections (v: vehicle, ONCOS-102 or ONCOS-204). **D.** Differences between change of CD107a expressing and cytokines producing (IL-2, TNF and IFNγ) CD4+ T cells induced by vehicle infected tumor cells. **E.** Differences between change of CD107a expressing and cytokines producing (IL-2, TNF and IFNγ) CD8+ T cells induced by vehicle (grey), ONCOS-102 (top, green) or ONCOS-204 (bottom, red) infected tumor cells. Friedman test with Dunn's correction was used to compare the groups of paired samples exposed to different conditions, n=8. *p<0.05, **p<0.01, ***p<0.001. Medians are represented.

Figure S3

Representative histogram of EGFR (**A**) and ICOSL (**B**) expressions on A375 and A549 tumor cell lines. Radar plot of the frequency of CD107a+, IFNγ, TNF+ and IL-2+ CD4+ (**C**) and CD8+ (**D**) T cells after co-culture with BsAb and A549 tumor cells exposed to different conditions (v: vehicle, ONCOS-102 or ONCOS-204). Friedman test with Dunn's correction was used to compare the groups of paired samples exposed to different conditions, n=3. *p<0.05. Medians are represented.

Figure S4

A. Different frequencies of IFNγ, TNF and MIP-1β positive conventional and γδ T cells upon suboptimal (0.5ng/ml) and high (10ng/ml) EGFRxCD3 BsAb concentration. γδ T cell (**B**) and subpopulation Vδ1 and Vδ2 (**C**) frequency. **D.** Surface expression of CD69 in γδ T cell after 48h incubation with target cells in different conditions. All data reported in **C-D** were evaluated after 48h incubation with target cells in the different conditions: presence or absence of BsAb and the different A375 tumor cell infections (v: vehicle, ONCOS-102 or ONCOS-204). **E.** Frequency of IFNγ, TNF and MIP-1β positive γδ T cells after 6h incubation with target cells in different conditions. **F.** Proportion of proliferative γδ T cells after 10 days of incubation with target cells in different conditions. Friedman test with Dunn's correction was used to compare the groups of paired samples exposed to different conditions, n=5. *$p<0.05$, **$p<0.01$. Medians are represented.

Figure S5

Surface ICOS expression (**A**) and frequency of CD107a and TNF positive (**B**) CD4+ (left) and CD8+ (right) T cells after 48h incubation with A375 target cells and BsAb in different conditions: transfection with vehicle or ICOSL plasmids **C.** Surface CD69 (top) and ICOS (bottom) expression on CD3+ T cells using 2 different anti-ICOS antibodies (grey: C398.4A and blue: Dx29) after 48h incubation with A375 tumor cell infected with different concentrations of ONCOS-204 (0, 5, 10, 100 vp/target cells). **D.** Control stimulation using surface CD69 expression on CD4+ (left) and CD8+ (right) T cells after 48h incubation with target cells in different conditions: presence or absence of BsAb and the different A375 tumor cell infections (v: vehicle, ONCOS-102 or ONCOS-204). **E.** Radar plot of the frequency of CD107a+, IFNγ and TNF+ CD4+ and CD8+ T cells after co-culture with A375 tumor cells infected with vehicle or A375 tumor cells and supernatant issue from vehicle-infected A375 cells. Student t test or Friedman test with Dunn's correction was used to compare the two conditions or groups of paired samples exposed to different conditions, n=3. *$p<0.05$. Medians are represented.
