# Supplementary figures and images for: Inducing expression of ICOS-L by oncolytic adenovirus to enhance tumor-specific bi-specific antibody efficacy
Source: J Transl Med. 2024 Mar 7;22:250. doi: 10.1186/s12967-024-05049-2 (PMC10921603; doi:10.1186/s12967-024-05049-2)

Figure S1

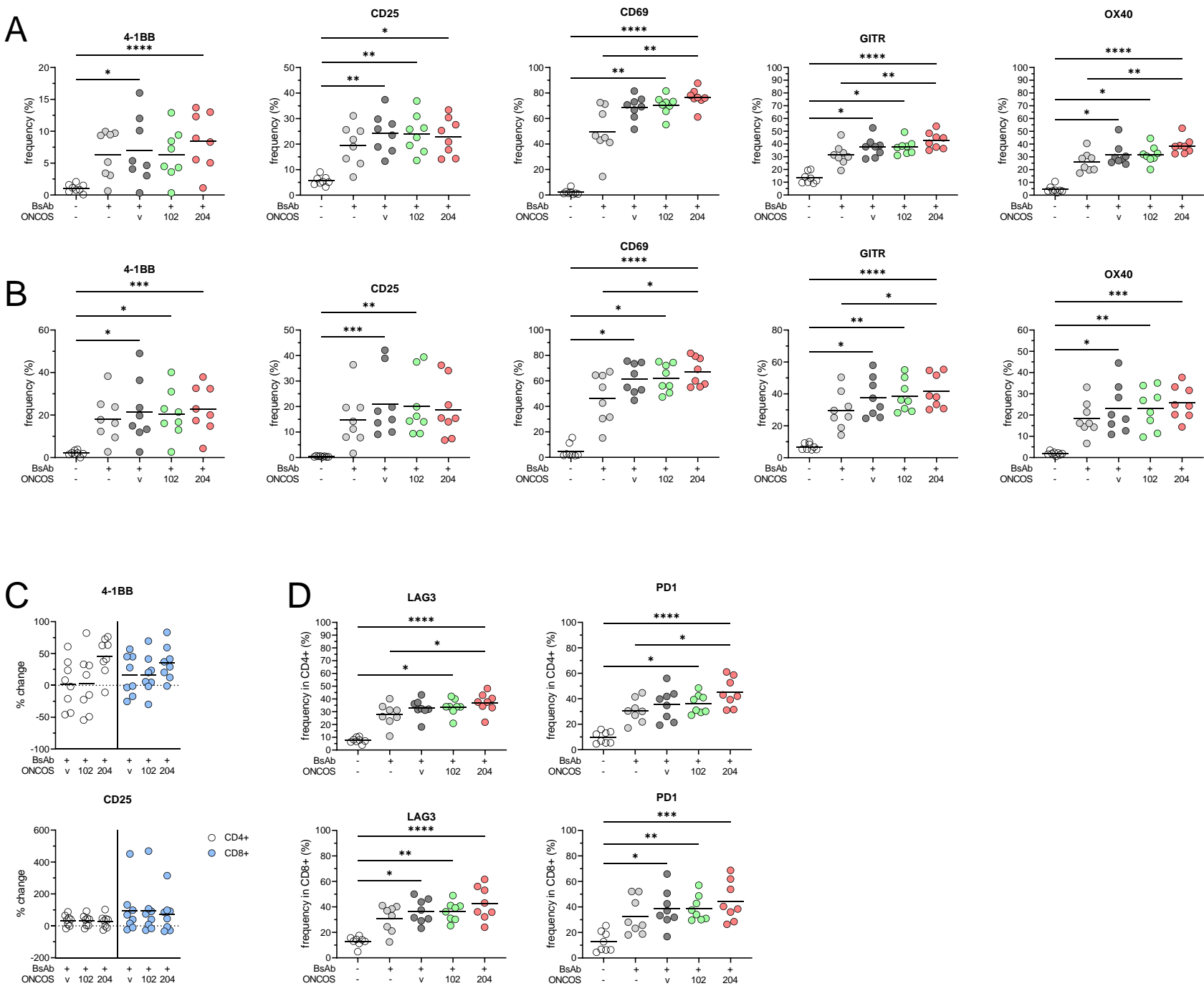

Figure S2

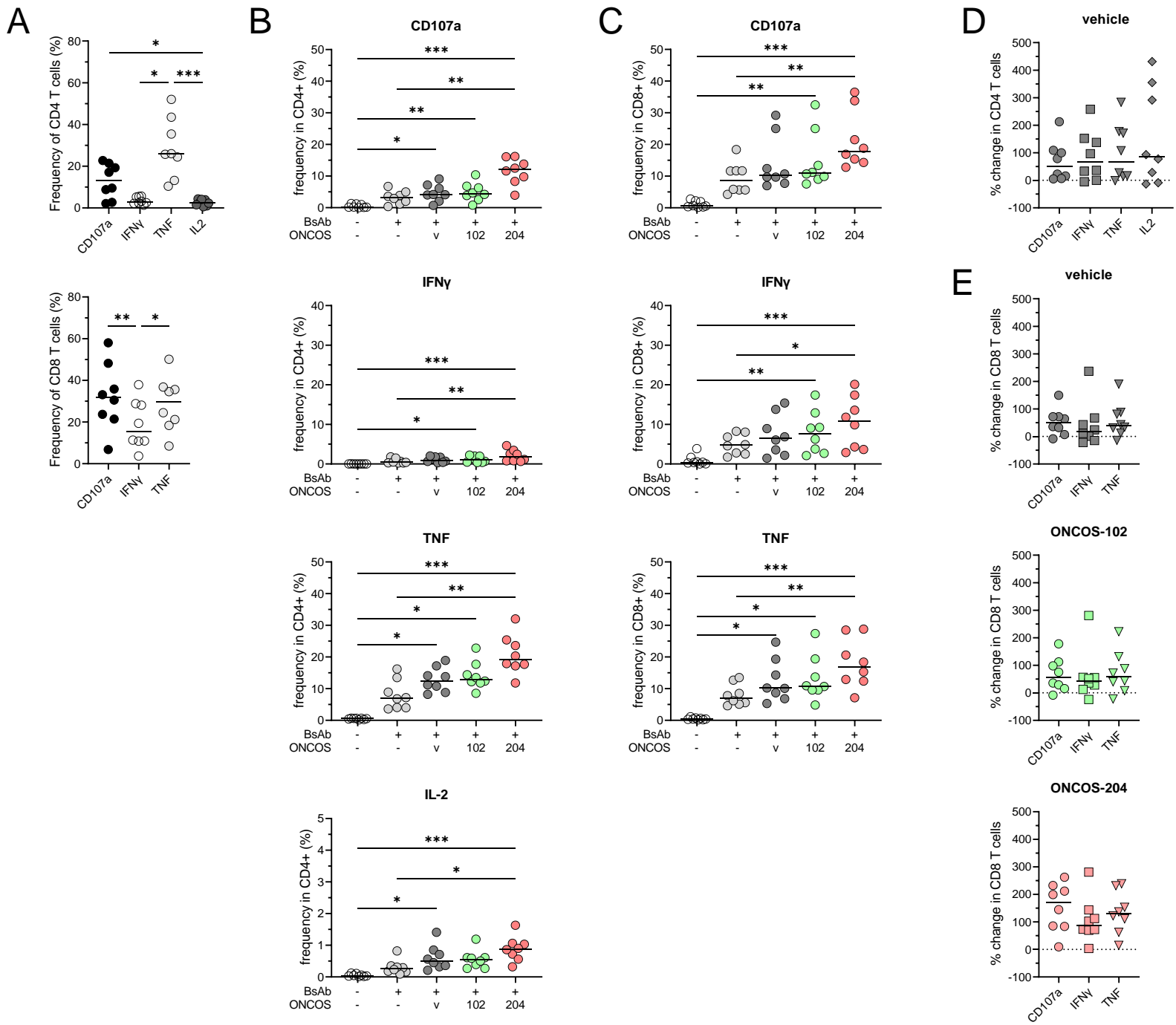

Figure S3

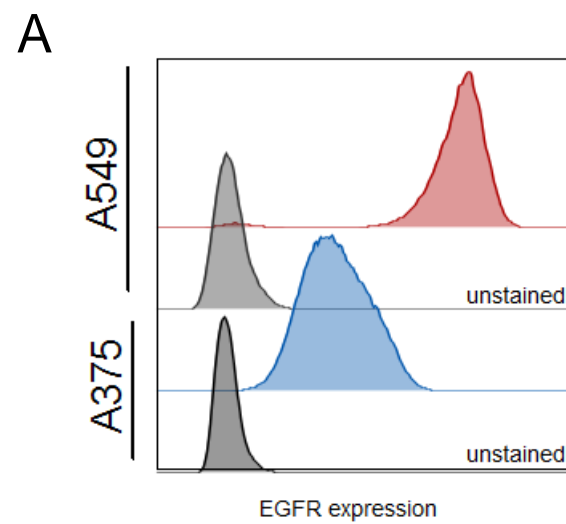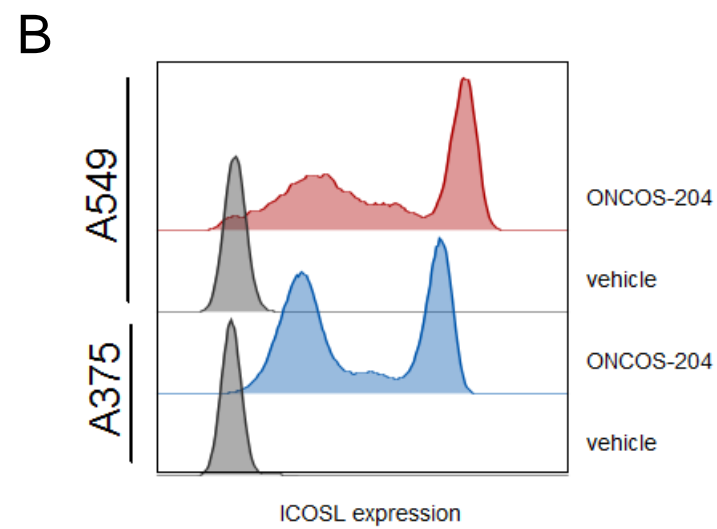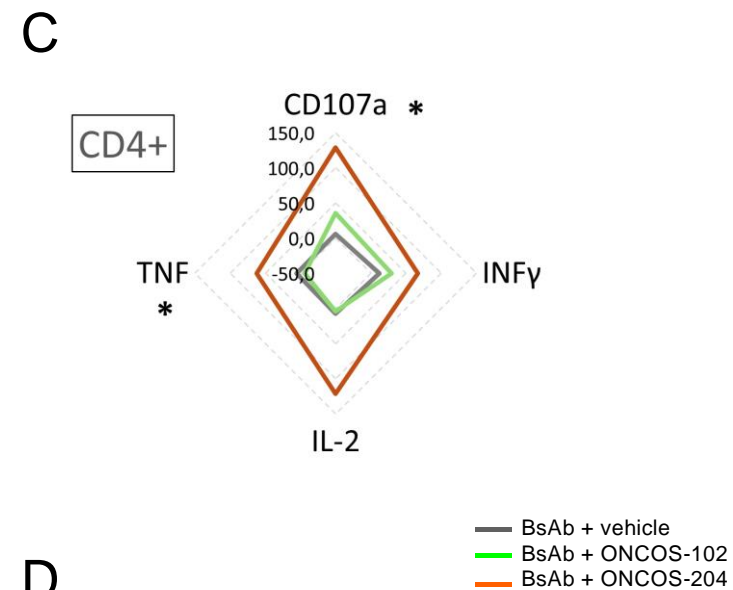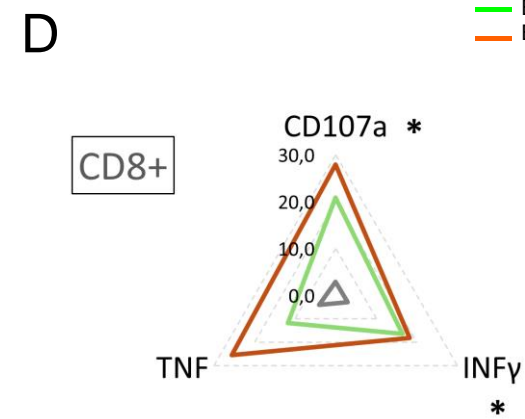

Figure S4

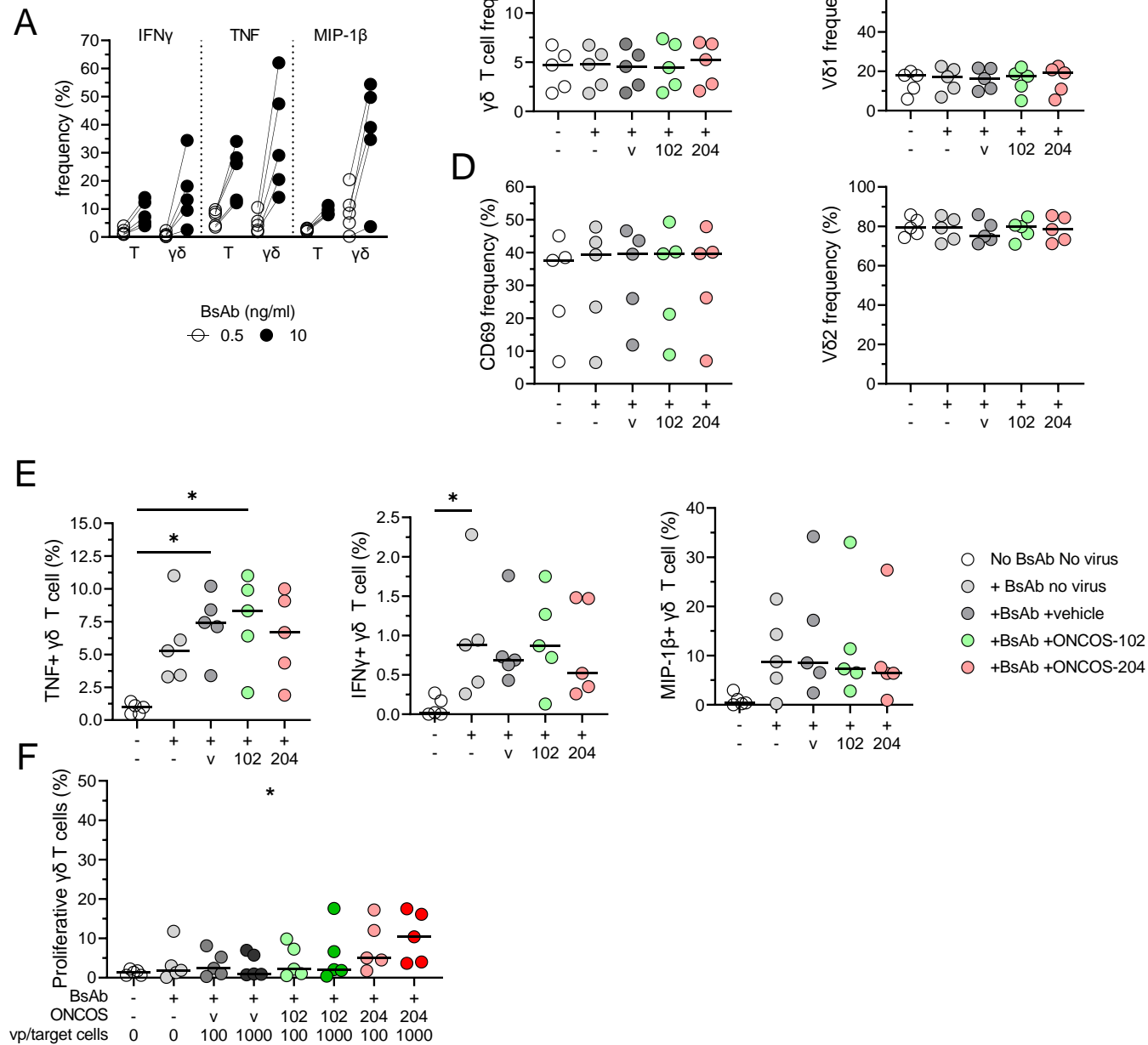

Figure S5

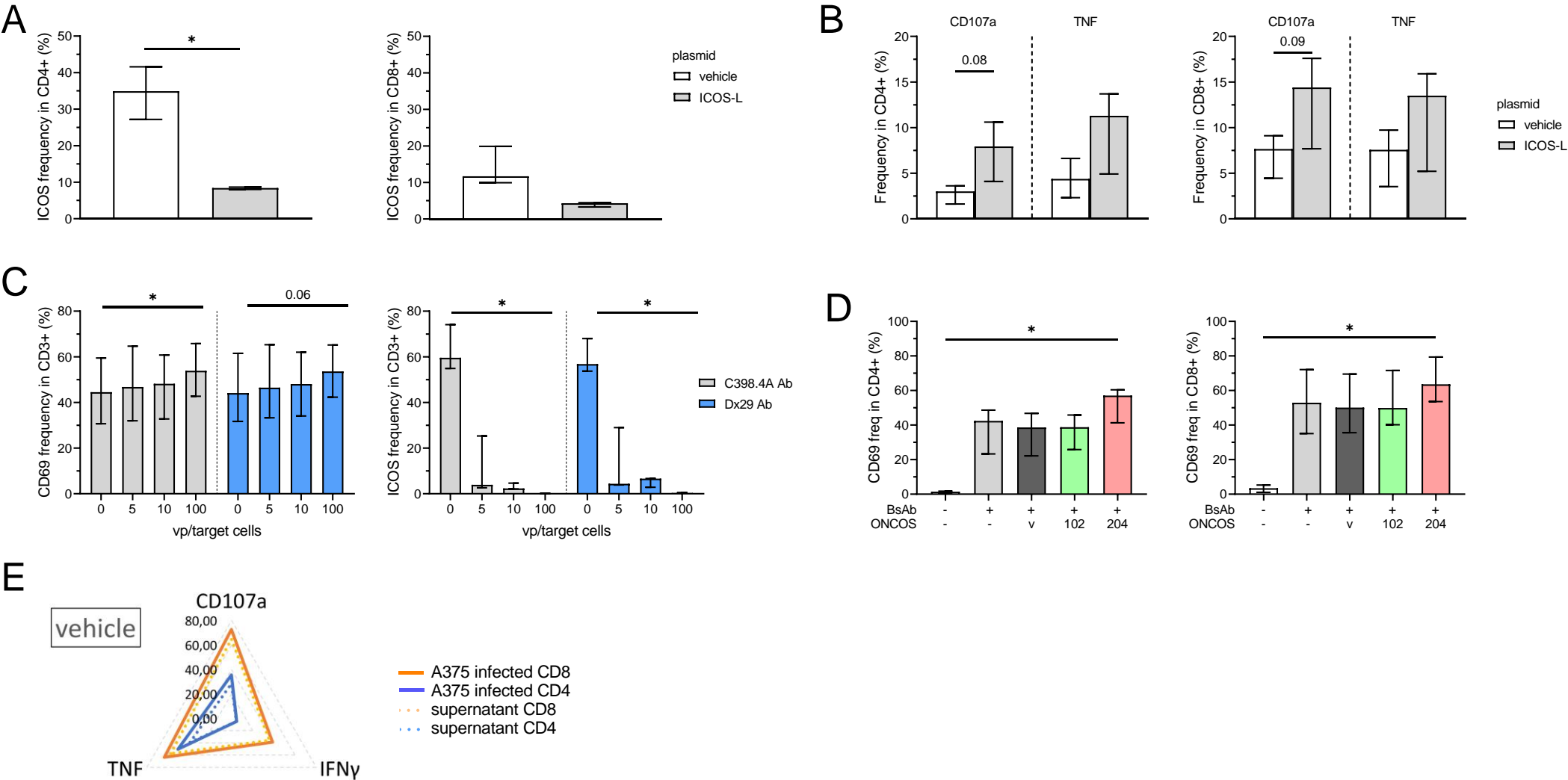

Supplement: Supplementary file 2 — Additional file 2. Supplementary Figures S1–S5 [file 12967_2024_5049_MOESM2_ESM.pdf]
